# Application rates to surgical residency programs in Canada

**DOI:** 10.36834/cmej.58444

**Published:** 2020-07-15

**Authors:** Todd Dow, Connor McGuire, Emma Crawley, Dafydd Davies

**Affiliations:** 1 Faculty of Medicine, Dalhousie University, Nova Scotia, Canada; 2 Division of Plastic Surgery, Department of Surgery, Dalhousie University, Nova Scotia, Canada; 3 Division of Paediatric General & Thoracic Surgery, Department of Surgery, Nova Scotia, Canada

## Abstract

**Purpose:**

The purpose of this study is to identify if the previously reported declining interest in surgery amongst medical students persists, and also to provide more descriptive analysis of trends by surgical specialty and medical school. Our hypothesis is that the previously reported decreasing interest in surgery remains constant for some surgical disciplines.

**Methods:**

The Canadian Resident Matching Service and the Association of Faculties of Medicine of Canada provided data for this study. Several metrics of interest in surgery, including overall application trends, applications by discipline, and rankings by school of graduation were evaluated. Descriptive statistics and linear regression modeling were used.

**Results:**

Between 2007 and 2017 the number of non-surgical residency positions and Canadian medical graduates increased significantly. However, the number of surgical residency positions and applications to surgical programs did not change significantly. The number of rankings to orthopedic and vascular surgery decreased significantly. Likewise, applicants to general, orthopedic, plastic, otolaryngology, and vascular surgery decreased significantly. Vascular surgery saw a significant decrease in first choice rankings. Total rankings to surgical programs increased significantly at McGill, with no significant change at other Canadian institutions.

**Conclusions:**

The findings of this study suggest that while the number of applicants to surgical residency positions has been consistent, it is not keeping pace with the growing number of both CMGs and non-surgical residency positions. Furthermore, by using other measures of medical student interest in surgical specialties, such as the total number of rankings to a specialty through the residency matching process, the total number of applicants applying to a surgical discipline and the total number of first choice ranks that each surgical discipline received, we have demonstrated that there is a possible declining interest in some surgical discipline

## Abstract (fr)

**Objectif:**

Cette étude vise à établir s’il persiste un déclin de l’intérêt pour la chirurgie parmi les étudiants en médecine, etfournir une analyse plus descriptive des tendances par spécialité chirurgicale et par faculté de médecine. Notre hypothèse est que le déclin précédemment rapporté de l’intérêt pour la chirurgie reste constant pour certaines disciplines chirurgicales.

**Méthodes:**

Le Service canadien de jumelage des résidents et l’Association des facultés de médecine du Canada ont fourni les données pour la présente étude. Plusieurs paramètres d’intérêt en chirurgie ont été évalués, dont les tendances globales des demandes d’admission, les demandes par discipline et les classements par faculté de diplomation. Nous avons eu recours à des statistiques descriptives et à une modélisation par régression linéaire.

**Résultats:**

Entre 2007 et 2017, le nombre de postes de résidence dans des spécialités non chirurgicales et de diplômés canadiens en médecine a augmenté de manière importante. Toutefois, le nombre de postes de résidence en chirurgie et de demandes d’admission pour ces programmes n’a pas varié de façon significative. Le nombre de classements pour la chirurgie orthopédique et vasculaire a diminué significativement. Le nombre de demandes d’admission en chirurgie générale, orthopédique, plastique, otorhinolaryngologique et vasculaire a également diminué significativement. On a aussi observé une baisse significative des classements comme premier choix pour la chirurgie vasculaire. Les classements totaux pour les programmes de chirurgie ont augmenté de manière importante à McGill, sans changement significatif dans les autres institutions canadiennes.

**Conclusions:**

Bien que le nombre de demandes d’admission à des postes de résidence en chirurgie ait été constant, les résultats de cette étude suggèrent qu’elles ne suivent pas le rythme du nombre croissant de DMC et de postes de résidence non chirurgicale. En outre, à l’aide d’autres mesures d’intérêt des étudiants pour les spécialités chirurgicales, comme le nombre total de classements pour une spécialité via le processus de jumelage des résidents, le nombre total de demandes d’admission àune discipline chirurgicale et le nombre total de classements comme premier choix pour chaque discipline chirurgicale, nous avons démontré qu’il existe un déclin possible de l’intérêt pour certaines disciplines chirurgicales.

## Introduction

The burden of chronic disease, combined with the aging population in North America is putting a strain on health care systems. As our population ages, it can be expected that the number of patients needing surgical treatments will increase in a similar fashion. Currently, surgically treated conditions are estimated to account for up to 30% of the global burden of disease.^1^ It is of the utmost importance that a proficient and talented surgical workforce is maintained to meet the population’s demands.

Despite this growing need, previous publications have suggested that in Canada and the United States applications to surgical residency programs have been *decreasing* for over a decade, despite *increasing* medical school enrollment.^2^^,^^3^^,^^4^ Peel and colleagues reported that applications ranking surgical disciplines as a first choice in Canada through the Canadian Resident Matching Service (CaRMS) decreased from 24.7% in 1998 to 17.2% in 2016.^4^ This declining trend has drawn concern from surgical program directors who recognize that they may attract fewer competitive medical students to their programs.^5^^,^^6^ There have been a wide range of proposed explanations for the declining number of medical students matriculating into surgical residencies, including lifestyle, overall interest, the duration of training, lack of early surgical exposure, availability of positions upon completion of training and more.^7^^-^^14^

While CaRMS publishes annual reports on the number of students applying and matching to residency positions across Canada, there are few published studies examining trends over a recent period of time. The existing studies have mainly focused on factors influencing medical students interest in surgery or have been specialty-specific in nature.^2^^,^^5^ Additional studies are essential to assess whether the current surgical workforce is adequate for the population’s need.

In light of the perceived downward trend in surgical residency applications and the lack of studies addressing this issue, we undertook this study to primarily investigate the surgical residency application trends across Canada from 2007 to 2017. The purpose of this study was to identify application trends to surgical specialities and provide a deeper descriptive analysis of these trends. Our hypothesis is that the previously reported decreasing interest in surgery remains constant for most surgical disciplines. Our hypothesis is that application rates by individual medical school did not change between 2007 and 2017.

## Methods

CaRMS is a third party organization responsible for facilitating the residency matching process between senior medical students and residency programs across Canada. All medical students who desire a residency position in Canada must use CaRMS to match to their desired program and school of training. There are two iterations of CaRMS. The first iteration is limited to only Canadian Medical Graduates (CMGs) who are able to apply to any residency program across the country. The second iteration is open to CMGs who did not match in the first iteration, foreign applicants and International Medical Graduates (IMGs) who can apply to the remaining vacant residency positions across Canada. Data analyzed in this study were from the first iteration of CaRMS, and thus only contain CMG data and not foreign applicants or IMGs. All CMGs who applied to surgical residency positions in Canada in the first iteration of CaRMS from 2007 to 2017 (inclusive) were included in this study. In collaboration with the research department of CaRMS, our team was provided yearly data on the total residency positions, the total non-surgical residency positions, the total surgical residency positions, the total number of applications each surgical discipline received the total number of applicants to each surgical discipline, the total number of first choice ranks each surgical discipline received, the total number of students who matched to each surgical discipline and the total number of applications to surgery each Canadian university had. Furthermore, data regarding the total number of medical school graduates across Canada from 2007 to 2017 were also examined using public data from The Association of Faculties of Medicine of Canada.^15^ All received data comply with privacy laws governing the CaRMS organization and their data usage policies.

We used various metrics to evaluate application trends to surgical residency positions. Our initial plan was to look at the proportion of total applicants to any surgical discipline compared to the total number of CMGs. This measurement would provide us with a yearly proportion of CMGs applying to surgery. To further investigate application rates by surgical discipline, we assessed the total number of applications (or “rankings”) each surgical discipline received yearly. Each student is able to rank numerous specialties and programs based on their preference and exposure to those programs through CaRMS, which acts as an application to that program. Therefore, students who rank multiple programs in one surgical field would be counted multiple times in the “total number of rankings” measurement for each specialty they applied to. To address this confounding factor and to clarify our results, we assessed the “total number of applicants” each surgical discipline received yearly. This metric quantifies the absolute number of students who ranked a discipline even once through CaRMS. This variable also presents a confounding factor in that if a student were to apply to more than one surgical discipline they would be counted as one “ applicant” for each program. Therefore, to clarify our results further, the total number of “first choice ranks” each surgical discipline received yearly. This metric quantifies the absolute number of students who ranked that discipline as their first choice through CaRMS. Each student would only have one “first choice” and therefore could not be counted multiple times.

In addition to application rates to surgery by discipline, we also examined the total number of rankings to surgical residency programs by the medical school of graduation. This provided a measure of that school’s desirability to train for careers in surgical specialties. The disciplines that were considered to be surgical and were assessed in this study include general surgery, orthopedic surgery, ophthalmology, vascular surgery, cardiac surgery, neurosurgery, plastic surgery, otolaryngology, and urology.

Descriptive statistics were used to evaluate all variables. Linear regression modeling was used to assess the change in application rate over time. The independent variable was the year of application and the dependent variables were the various metrics for measuring interest in surgery as described above. Linear regression modeling was conducted using data over the 10-year period (2007 to 2017) as well as the most recent 5 years (2013 to 2017). Linear regression results were reported in p-values for assessing significance along with unstandardized beta coefficients and adjusted R square values for testing the strength of association between time and application trends. A beta coefficient describes the strength of association between the dependent to the independent variable. If a beta coefficient is positive, then the interpretation is that for every 1-unit increase in the independent variable (year) the dependent variable (number of applications) will increase by the beta coefficient value. If negative, then for every 1-unit increase in year then the number of applications will decrease by the beta coefficient value. SPSS version 24 was used for all analyses (IBM SPSS Statistics, Armonk, NY, US). A p-value of less than or equal to 0.05 (two-sided) was considered significant. Research ethics board approval was not necessary for this study as all data analyzed is available to the public.

## Results

**Figure 1:**
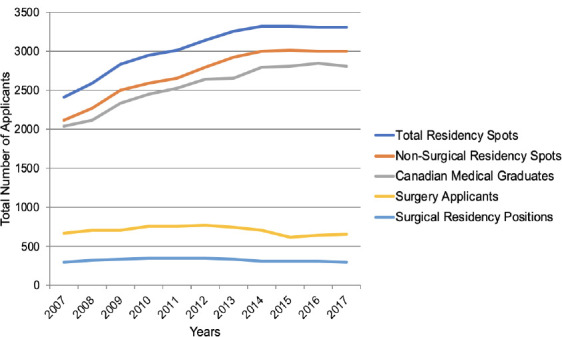
The total number of residency positions, non-surgical and surgical residency positions, Canadian medical graduates, and surgery applicants from 2007 to 2017 in Canada.

The total number of residency positions in Canada increased significantly by 26.9% over the ten year period, from 2417 spots in 2007 to 3305 residency spots in 2017 (*p* < 0.01). The total number of non-surgical residency positions increased significantly by 29.6%, from 2114 to 3003 (*p* < 0.01, (R) = 0.93, adjusted R^2^ = 0.89), while the total number of surgical residency positions remained constant, decreasing only by 0.3%, from 303 to 302 (*p* = 0.28, (R) = -0.36, adjusted R^2^ = 0.03). The total number of CMGs increased by 27.2%, from 2046 to 2811 (*p* < 0.01, (R) = 0.96, adjusted R^2^ = 0.91), while the total number of surgery applicants remained fairly consistent, only decreasing by 2.6%, from 674 to 657 over the same time period (*p* = 0.21, (R) = -0.42, adjusted R^2^ = 0.08). Due to the substantial increase in CMGs, the proportion of applicants to surgery decreased from 32.9% to 23.4% despite the total number of applicants to surgery remaining constant (Figure 1).

**Figure 2:**
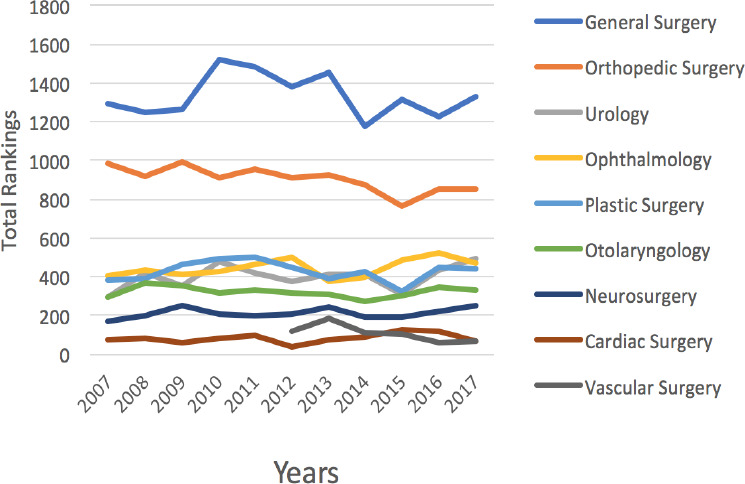
The total number of rankings by surgical discipline from 2007 to 2017 in Canada. All lines in black denote significant changes in the total number of rankings.

The total number of rankings by surgical discipline are presented in Figure 2. Over the past 10 years from 2007 to 2017, the total number of rankings to orthopedic and vascular surgery decreased significantly by 15.7%, from 987 to 853 (*p* < 0.01, (R) = -0.78, adjusted R^2^ = 0.57) and 42.9%, from 121 to 69 (*p* = 0.03, (R) = -0.77, adjusted R^2^ = 0.49), respectively. There was no significant change in the number of ranks to general surgery (*p* = 0.71, (R) = -0.13, adjusted R^2^ = -0.9), urology (*p*=0.26, (R) = 0.37, adjusted R^2^ = 0.04), ophthalmology (*p* = 0.14, (R) = 0.48, adjusted R^2^ = 0.14), plastic surgery (*p* = 0.84, (R) = -0.07, adjusted R^2^ = -0.11), otolaryngology (*p* = 0.55, (R) = -0.21, adjusted R^2^ = -0.06), neurosurgery (*p* = 0.29, (R) = 0.35, adjusted R^2^ = 0.03), or cardiac surgery (*p* = 0.23, (R) = 0.4, adjusted R^2^ = 0.067; Figure 2).

**Figure 3:**
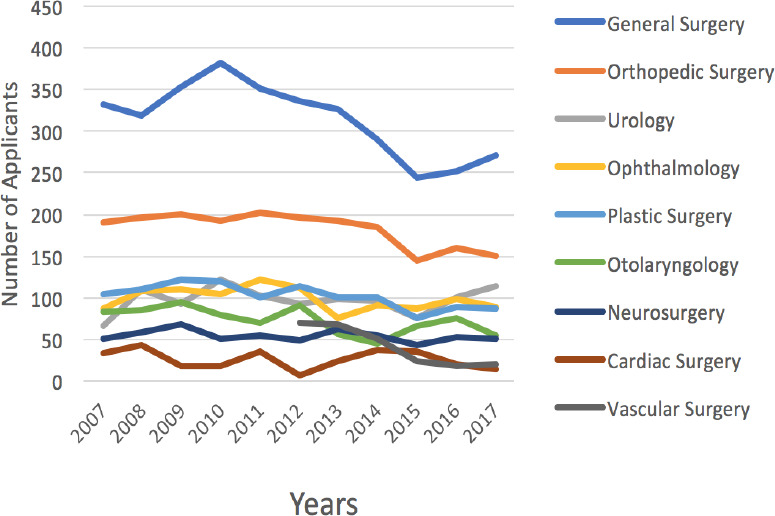
The number of applicants by surgical discipline from 2007 to 2017 in Canada. All lines in black denote significant changes in the number of applicants.

The number of applicants by surgical discipline are presented in Figure 3. Over the past 10 years from 2007 to 2017, the number of applicants decreased significantly n general surgery by 18.6%, from 333 to 271 (*p* < 0.01, (R) = -0.76, adjusted R^2^ = 0.53), orthopedic surgery by 0.9%, from 191 to 151 (*p* < 0.01, (R) = -0.78, adjusted R^2^ = 0.56), plastic surgery by 15.4%, from 104 to 88 (*p* = 0.01, (R) = -0.73, adjusted R^2^ = 0.49), otolaryngology by 33.7%, from 83 to 55 (*p* = 0.03, (R) = -0.67, adjusted R^2^ = 0.38), and vascular surgery by 71.4%, from 70 to 20 (*p* < 0.01, (R) = -0.95, adjusted R^2^ = 0.87). There was no significant change in the number of applicants to urology (*p* = 0.58, (R) = 0.19, adjusted R^2^ = -0.07), ophthalmology (*p* = 0.26, (R) = -0.37, adjusted R^2^ = 0.04), neurosurgery (*p* = 0.24, (R) = -0.39, adjusted R^2^ = 0.05), or cardiac surgery (*p* = 0.39, (R) = -0.29, adjusted R^2^ = -0.08; Figure 3).

**Figure 4:**
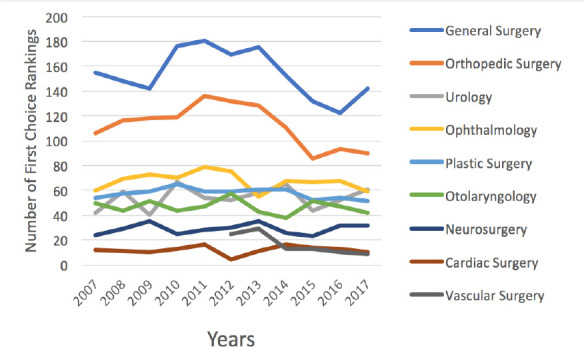
The number of first choice rankings by surgical discipline form 2007 to 2017 in Canada. All lines in black denote significant changes in the number first choice rankings.

The number of first choice rankings by surgical discipline are presented in Figure 4. Over the past 10 years from 2007 to 2017, there was a significant decrease of 64%, from 25 to 9 in the number of first choice rankings to vascular surgery (*p* = 0.04, (R) = -0.87, adjusted R^2^ = 0.7). There was no statistically significant change in the number of first choice rankings to other specialties (Figure 4).

Only McGill University saw its students significantly increase their total number of rankings to surgical residency programs, by 37.3% (*p* = 0.03, (R) = 0.67, adjusted R^2^ = 0.45).

## Discussion

This study demonstrates that even though the total number of residency positions, non-surgical residency positions, and CMGs have increased over the past decade, the total number of surgical residency positions and surgery applicants has remained consistent. Provincial Health Ministers may be pleased with these results, as most of the residency positions were added to address the shortage of family physicians. However, this trend should be monitored to ensure that an adequate number of high-quality surgeons continue to be trained and there are no untoward effects on the surgical workforce. Furthermore, these trends result in proportionally fewer CMGs going into surgery. Traditionally, the proportion of applicants to surgical disciplines has been used to measure interest. If we were to follow that same trend and use proportion as a measure of interest, then our study would agree with the historical data that interest in surgical disciplines is decreasing, despite the absolute number of applicants remaining constant.

We argue that if the absolute number of applicants is remaining consistent, then perhaps other avaiable variables should be assessed to better understand student interest in surgery. To further assess interest in surgical disciplines, this study utilized three other available variables: the total rankings by surgical discipline, the total number of applicants to each surgical discipline and the total number of first choice ranks that each surgical discipline received. This was done to address some of the hidden biases in the available data and ideally to paint a clearer picture of whether overall interest in surgery is declining. Upon assessment of this data, it became apparent that over the 10 year period of this study urology, ophthalmology, neurosurgery, and cardiac surgery were the only specialties that did not have a significant change in any of the variables of interest that we assessed (total number of rankings, the total number of applicants or the total number of first choice ranks).

Interestingly, our data from 2007 to 2017 shows that vascular surgery was the only specialty that had a statistically significant decrease in all three of our interest variables: total number of rankings, the total number of applicants, and the total number of first choice rankings. As vascular surgery only became its own recognized residency program in 2012 in Canada, the trends we have identified are likely due to the initial influx of interest in a new program that has now subsided. The interest in vascular surgery amongst medical students should be closely evaluated and monitored. However, the trends we have identified may have plateaued as the last 3 years have had fairly consistent numbers.

For the discipline of orthopedic surgery, we identified that from 2007 to 2017 the total number of rankings and the total number of applicants decreased significantly. However, the total number of first choice ranks did not change significantly. The Royal College of Physicians and Surgeons of Canada surveyed orthopedic surgeons from 2011 to 2017 and reported that as of July 1, 2017, 165 recent graduates were still seeking full-time employment (this includes those completing locums, fellowships or research)^16^. Likewise, an average of 36% of orthopedic surgeons reported not having a job at the time of their certification^16^. This report also indicated that for every 9 orthopedic surgeons under the age of 35 there are 10 who are 65+ years old.^16^ Lastly, they reported that fewer orthopedic surgeons were entering the training pipeline and between 2011 and 2015 there was a 9% average decrease in the number of residency spots and new trainees in the discipline^16^. These findings suggest that Canada suffers from a stagnant workforce of orthopedic surgeons that does not accommodate recent graduates. This information has likely influenced medical student’s career decisions and could be a contributing factor to the decrease in the total rankings and the total number of applications that have been identified over the past 10 years.

General surgery, plastic surgery, and otolaryngology all saw a statistically significant decrease in the total number of applicants between 2007 and 2017. The decrease in the number of applicants to these surgical specialties does not seem to be supported by the same limited occupational opportunities as orthopedic surgery. Therefore, the decrease in total applicants could be due to a true decrease in interest amongst medical students. Each of these three specialties are commonly identified as “competitive”, which could be acting as an increasingly stronger deterrent amongst medical students. In 2019, there were a reported 0.48 residency positions for each applicant to plastic surgery, 0.70 residency positions for each applicant to otolaryngology and 0.85 residency positions for each applicant to general surgery.^17^ Interestingly, other specialties such as ophthalmology, urology, and neurosurgery have similar reported available residency positions but did not see the same decrease in total applicants. Therefore, each of these specialties should carefully monitor whether these trends have downstream effects on attrition and performance throughout the program. However, the data suggests that despite the decrease in applicants, the programs will not be subject to a shortage of surgeons as these programs continue to fill their first year-residency positions each year.

In their discussion paper, Austin and Wanzel investigated surgical application trends from 2002 to 2013. They found that the number of medical students selecting a surgical specialty as their first choice discipline decreased from 20.8% in 2002 to 13.0% in 2014.^2^ They also felt that applicants to surgical disciplines were likely becoming less competitive as the number of vacant seats in surgical programs across Canada was rising during that period. They hypothesized that both the number and the quality of applicants were decreasing due to less time devoted to core surgical rotations during clerkship, fewer surgery specific lectures in medical school and students increasingly opting for more “lifestyle friendly” specialties.^2^ Our study does not assess the quality of applicants applying to surgical residency positions. However, our findings do echo their sentiment that despite increased numbers of medical school graduates, applications to surgical programs remain stagnant. Additionally, our study further breaks down this issue; showing that application rates by both schools of graduation and specific surgical subspecialties are consistently low compared to increasing medical school enrollment rates.

While our study has evaluated the quantitative aspect of applications to surgical residency programs, our data do not examine the factors influencing these trends. Studies of lower and upper year medical students have shown that students who are younger, male, single, hospital oriented, and more interested in the prestige of their profession are more likely to pursue surgery.^5^^,^^18^^,^^19^ A recent systematic review of factors that affect the choice of pursuing a career in surgery demonstrated three distinct core concepts that impact decision making.^4^ These core concepts were: gender, features of medical school surgical education, and integration into the perceived culture of surgery.^4^ Gender discrimination, lack of gender-specific role models, and the impact of a surgical lifestyle (including poor support for childcare and maternity leave) were all factors related to decreased interest in a surgical career. The two main factors during medical school that had a *positive* impact on interest in pursuing a surgical career were early surgical exposure before clerkship and exposure to surgical simulations.^4^ While a number of these factors are difficult to modify, early surgical exposure in medical school and improving gender-based issues are potential areas that may be suitable for targeted interventions. Currently, Medical schools are progressively modifying their clerkship structure to accommodate teaching and other academic commitments. While these modifications are heavily evaluated, the administration should ensure they are not restricting the limited amount of surgical exposure available to students.

With a growing and aging population, recent studies in the United States have addressed the possibility of an impending shortage in the surgical workforce.^20^ This issue is largely understudied in Canada and what research exists is outdated. A brief review of the literature reveals orthopedic surgery, cardiac surgery, and pediatric general surgery have made predictions regarding their forthcoming workforce shortfalls.^21^^-^^23^ However, no comprehensive study has evaluated the Canadian surgical workforce as a whole.^21^^-^^23^ Workforce data from the Royal College of Physicians and Surgeons of Canada (RCPSC) indicates that as of 2015, the balance of surgeons retiring was approximately equal to the number of new graduating residents.^24^ Data indicate that the surgical workforce is slightly older (17% aged 65 and above) compared to all other specialties (14% aged 65 and above). This suggests that although the number of residency graduates is currently replacing the retiring surgeon population, the older surgical workforce may see an increase in retirement in the near future. There is also evidence of limited job opportunities in some surgical specialties.^25^ These limited job opportunities combined with an aging population could create an environment where the practicing surgical workforce may not meet the needs of the population. Interestingly, the World Health Organization has recently published evidence that the global burden of surgical disease is projected to *increase* significantly in the coming decades.^1^^,^^26^

The main strengths of this study include using surgical subspecialty specific data that is recent and up to date, the use of a reliable, validated and complete database, and the use of multiple measures of application rates. We feel that understanding these trends remains important to maintaining the quality of applicants to surgical specialties in Canada. The limitations of this study are the confounding factors of some of the interest variables we assessed. For “total number of ranks,” one student could rank multiple surgical specialties multiple times. Likewise, “total number of applicants to each surgical specialty” is confounded by that fact that one student may apply to more than one surgical specialty and thus be counted as an applicant for each. Furthermore, the inability to capture demographics of students for a more in-depth analysis of the predictive factors in application rates to surgical residency programs, the limited information from student’s perspective and the limited information on occupational workforce trends and government funding for residency positions year to year. In the future, further research must be conducted to understand the specific factors affecting not only specialty choice but also success in the chosen specialty for Canadian medical school graduates. This research is necessary to ensure a strong, adequate surgical workforce in the future.

## Conclusions

The findings of this study suggest that while the total number of applicants to any surgical residency positions has been consistent, it is not keeping pace with the growing number of both CMGs and non-surgical residency positions. Furthermore, by using other measures of medical student interest in surgical specialties, such as the total number of rankings to a specialty through the residency matching process, the total number of applicants applying to a surgical discipline and the total number of first choice ranks that each surgical discipline received, we have demonstrated that there is a possible declining interest in some surgical disciplines.
